# Proteomic Analysis of the Mammalian Katanin Family of Microtubule-severing Enzymes Defines Katanin p80 subunit B-like 1 (KATNBL1) as a Regulator of Mammalian Katanin Microtubule-severing* [Fn FN2]

**DOI:** 10.1074/mcp.M115.056465

**Published:** 2016-02-29

**Authors:** Keith Cheung, Silvia Senese, Jiaen Kuang, Ngoc Bui, Chayanid Ongpipattanakul, Ankur Gholkar, Whitaker Cohn, Joseph Capri, Julian P. Whitelegge, Jorge Z. Torres

**Affiliations:** From the ‡Department of Chemistry and Biochemistry, University of California, Los Angeles, California, 90095;; §Pasarow Mass Spectrometry Laboratory, The Jane and Terry Semel Institute for Neuroscience and Human Behavior, David Geffen School of Medicine, University of California, Los Angeles, California 90095;; ¶Molecular Biology Institute, University of California, Los Angeles, California, 90095;; ‖Jonsson Comprehensive Cancer Center, University of California, Los Angeles, California, 90095

## Abstract

The Katanin family of microtubule-severing enzymes is critical for remodeling microtubule-based structures that influence cell division, motility, morphogenesis and signaling. Katanin is composed of a catalytic p60 subunit (A subunit, KATNA1) and a regulatory p80 subunit (B subunit, KATNB1). The mammalian genome also encodes two additional A-like subunits (KATNAL1 and KATNAL2) and one additional B-like subunit (KATNBL1) that have remained poorly characterized. To better understand the factors and mechanisms controlling mammalian microtubule-severing, we have taken a mass proteomic approach to define the protein interaction module for each mammalian Katanin subunit and to generate the mammalian Katanin family interaction network (Katan-ome). Further, we have analyzed the function of the KATNBL1 subunit and determined that it associates with KATNA1 and KATNAL1, it localizes to the spindle poles only during mitosis and it regulates Katanin A subunit microtubule-severing activity *in vitro*. Interestingly, during interphase, KATNBL1 is sequestered in the nucleus through an N-terminal nuclear localization signal. Finally KATNB1 was able to compete the interaction of KATNBL1 with KATNA1 and KATNAL1. These data indicate that KATNBL1 functions as a regulator of Katanin A subunit microtubule-severing activity during mitosis and that it likely coordinates with KATNB1 to perform this function.

The remodeling of microtubule structures is important for many aspects of cell physiology including cell division, motility, morphogenesis, and signaling (1). A major group of proteins that remodel microtubules through microtubule-severing activities are the AAA ATPase containing proteins that include Spastin, Fidgetin, and the Katanins (1–4). Katanins are composed of a catalytic p60 subunit (A subunit, which contains the AAA ATPase domain) and a regulatory p80 subunit (B subunit) (2). However, i*n-vitro* biochemical studies have shown that the A subunit can form an unstable 14–16 nm hexameric ring structure, which can sever microtubules in the presence or absence of the B subunit (2, 5, 6), indicating that the A subunit does not require the B subunit for microtubule-severing activity. However, the B subunit has been shown to regulate the rate of microtubule-severing by the A subunit (7, 8), hence its designation as a regulatory subunit. The Katanins have been implicated in the regulation of multiple processes that are important for cellular homeostasis, proliferation, and invasion including the severing of spindle microtubules to generate microtubule density during meiosis and mitosis, the severing of the cilia axoneme microtubules during ciliary resorption, the severing of intercellular bridge microtubules during cytokinesis and the severing of cytoplasmic microtubules to promote cell morphogenesis and migration (5, 9–13). For example, in *Caenorhabditis elegans*, the microtubule-severing activity of the Katanin A subunit MEI-1 is required for regulating spindle length and density during meiotic spindle formation and for stabilizing the association of the meiosis I spindle to the oocyte cortex (14–16). Additionally, inactivation of a temperature sensitive MEI-1 mutant during metaphase disrupts bipolar spindle assembly and the alignment of chromosomes to the metaphase plate (17). In *Drosophila melanogaster*, the microtubule-severing activity of the Katanin p60-like 1 (Kat-60L1) A subunit is critical for neuronal development (18). Kat-60L1 mutants exhibit a reduced number and length of dendrites in neurons and a diminished neuronal responsiveness to chemical and thermal stimuli (19). Additionally, during mitosis the Katanin p60 (DmKat-60) A subunit localizes to chromosomes and facilitates microtubule depolymerization during chromosome segregation (4). In other organisms such as *Tetrahymena thermophila*, *Chlamydomonas reinhardtii, and Trypanosoma brucei*, Katanin A subunits are important for flagellar biogenesis and cell division (11, 20–22). Finally, the microtubule-severing activity of Katanin A subunits in *Arabidopsis thaliana* are critical for regulating cell specification, cell growth and cell wall biosynthesis (23–29).

Although the majority of Katanin studies have focused on understanding the function of the canonical p60 and p80 subunits in lower organisms, many organisms encode additional p60-like and p80-like proteins known as Katanin-like proteins. For example, the human genome encodes two alternatively spliced isoforms of the canonical p60 A subunit (KATNA1 chromosomal locus 6q25.1, protein IDs NP_001191005.1 and NP_008975.1) and two additional p60-like proteins (KATNAL1 chromosomal locus 13q12.3, protein ID NP_115492.1; KATNAL2 chromosomal locus 18q21.1, protein ID NP_112593.2). Similarly, in addition to the canonical p80 B subunit (KATNB1 chromosomal locus 16q21, protein ID NP_005877.2), the human genome encodes an additional p80-like protein (KATNBL1 chromosomal locus 15q14, protein ID NP_078989.1) (30). Interestingly, all human Katanin subunits are ubiquitously expressed across most tissue types including brain, lung, kidney, liver, pancreas, and skin and in established cell lines like HeLa cells (31). Human Katanins have important roles in regulating microtubule-dependent processes like mitotic spindle length and structure during cell division and mutation of Katanin subunits has been linked to human disorders like cerebral cortical malformation and male infertility (14, 32–35). Domain analyses of the human Katanin subunits indicate that KATNA1 and KATNAL1 share a similar domain architecture with an N-terminal microtubule interacting and trafficking domain (MIT)^1^ followed by a coiled coil domain (CC), a AAA ATPase domain (AAA) and a C-terminal VPS4 domain (VPS4_C) (36) (Fig. 1*A*). In contrast, KATNAL2 only contains the AAA domain, lacks the MIT, CC and VPS_4 domains, and has an N-terminal LisH (LIS1 homology) domain (Fig. 1*A*). Because KATNAL2 harbors a AAA domain that is required for Katanin microtubule-severing activity, it is predicted to have a role in microtubule-severing, however this has yet to be tested. Additionally, KATNAL2 lacks the MIT domain that is important for microtubule binding in other Katanin A subunits and whether the MIT domain is critical for microtubule-severing activity remains to be determined (37). Although KATNB1 and KATNBL1 both harbor a conserved C-terminal region (con80), only KATNB1 contains additional N-terminal proline-rich (Pro-rich) and WD40 domains that are absent in KATNBL1 (8, 30) (Fig. 1*A*). Interestingly, full length KATNB1 and the KATNB1 con80 domain alone have been shown to stimulate KATNA1 microtubule-severing activity, whereas the KATNB1 WD40 domain alone inhibits microtubule-severing (7, 8). However, the underlying mechanism of how the B subunits regulate the activity of the A subunits is still unclear. Additionally, outside of KATNA1 and KATNB1, the remaining human Katanin subunits remain poorly characterized.

Our previous proteomic analyses of human mitotic microtubule co-purifying proteins identified a then hypothetical p60-like protein KATNAL1 (38). Tandem affinity purification and mass proteomic analysis of KATNAL1 identified KATNA1, KATNAL1, KATNB1 and a then hypothetical p80-like protein C15orf29 (KATNBL1) as interactors, indicating that in humans multiple Katanins were likely involved in microtubule-severing (39). Consistent with this idea, depletion of human KATNA1 or KATNAL1 alone leads to mild changes in spindle size and mitotic defects (35, 40), indicating that multiple Katanins may be involved in regulating spindle size in mammals and/or that other microtubule-severing activities are able to compensate in the absence of either Katanin. Additionally, whether KATNAL2 has microtubule-severing activity that can compensate for the absence of other Katanin A subunits remains to be determined. Finally, the effect, if any, that KATNBL1 has on Katanin A subunit microtubule-severing activity also remains to be determined. To better understand the human Katanins and more broadly the mechanisms controlling mammalian microtubule-severing, we analyzed the human Katanin interactome (Katan-ome) through biochemical tandem affinity purifications and mass proteomic analyses. We further focused on the characterization of the poorly understood KATNBL1 subunit and its role in microtubule-severing through binding assays, microtubule-severing assays, competition binding assays, and subcellular localization studies. Our results showed that KATNBL1 is uniquely sequestered to the nucleus during interphase and associates with spindle poles in mitosis, is a regulator of KATNAL1 microtubule-severing activity, and competes with KATNB1 for binding to KATNA1 and KATNAL1. These results indicate that in humans microtubule-severing is complex and likely regulated by the concerted action of KATNB1 and KATNBL1.

## EXPERIMENTAL PROCEDURES

### 

#### 

##### Cell Culture and Cell Cycle Synchronization

All chemicals were purchased from Thermo Scientific (Waltham, MA) unless otherwise noted. HeLa and HeLa Flp-In T-REx LAP-tagged stable cell lines were grown in F12:DMEM 50:50 medium (Thermo Scientific) with 10% FBS, 2 mm l-glutamine and antibiotics, in 5% CO_2_ at 37 °C. Cells were induced to express the indicated LAP-tagged proteins by the addition of 0.2 μg/ml doxycycline (Sigma-Aldrich, St. Louis, MO). For synchronization of cells in mitosis, cells were treated with 100 nm Taxol (Sigma-Aldrich) for 16 h.

##### Plasmids, Mutation, and Generation of LAP-tagged Katanin Inducible Stable Cell Lines

For full-length *KATNA1*, *KATNAL1*, *KATNAL2*, *KATNB1*, and *KATNBL1, or STARD9-START and STARD9-MD* expression, cDNA corresponding to the full-length open reading frame of each Katanin or the indicated *STARD9* domains was fused to the C terminus of either HA (pCS2-HA-DEST vector), Myc (pCS2-Myc-DEST vector), FLAG (pCDNA3-FLAG-DEST vector), GST (pGEX-6p-1-DEST vector), or EGFP (pGLAP1 vector) using the Gateway cloning system (Invitrogen, Carlsbad, CA) as described previously (38). The pGLAP1-Katanin vectors were used to generate doxycycline inducible HeLa Flp-In T-REx LAP-KATNA1, KATNAL1, KATNAL2, KATNB1, and KATNBL1 stable cell lines that express the fusion protein from a specific single locus within the genome as described previously (39). For KATNBL1 mutations, pGLAP1-KATNBL1 was mutated using the QuickChange Lightning Site-Directed Mutagenesis Kit (Agilent Technologies, Santa Clara, CA) with primers carrying the desired mutations, as described previously (41), whereas truncation mutants were generated by PCR amplification and cloning into pGLAP1 as described above. All primers were purchased from Fisher Scientific. For a list of primers used see supplemental Table S3.

##### Immunoprecipitations

For cell extract immunoprecipitations (IPs), LAP-KATNA1, LAP-KATNAL1, LAP-KATNB1, or LAP-KATNBL1 HeLa stable cells lines were transfected with the indicated HA-tagged Katanin subunit expression vectors for 24 h and whole cell extracts were prepared in LAP150 lysis buffer (50 mm Tris pH 7.4, 150 mm KCl, 1 mm EDTA, 1 mm MgCl_2_, 10% glycerol) plus 250 μm ATP, 0.3% Nonidet P-40, 0.5 mm DTT and protease and phosphatase inhibitor mixture (Thermo Scientific). Cell extracts were cleared by centrifugation at 15K RPM for 10 min. One hundred forty micrograms of cleared lysate was incubated with 5 μl packed bead volume of anti-HA antibody conjugated magnetic beads (MBL, Woburn, MA) for 1 h at 4 °C. The beads were then washed 3 times with 50 μl of LAP150 lysis buffer and bound proteins were eluted with 20 μl of 1× Laemmli SDS sample buffer (Bio-Rad, Irvine, CA). Six percent of the sample inputs, 6% of the unbound fractions and the entire eluates from the immunoprecipitations were resolved on a 10% Tris gel (Bio-Rad) with Tris-Glycine SDS running buffer, transferred to a Immobilon PVDF membrane (EMD Millipore, Billerica, MA), immunoblotted with the indicated antibodies, and imaged with a LI-COR Odyssey imager (LI-COR Biosciences, Lincoln, NE).

##### In Vitro Binding Assays

For *in vitro* binding assays, HA, Myc, or FLAG-tagged KATNA1, KATNAL1, KATNAL2, KATNB1, or KATNBL1 were *in vitro* transcribed and translated (TnT® Quick Coupled Transcription/Translation System, Promega, Madison, WI) in 50 μl reactions. Two different Katanin reactions were combined and incubated with 5 μl packed bead volume of anti-HA antibody conjugated magnetic beads (MBL) for 1 h. Beads were washed four times with a wash buffer containing 10 mm Tris pH 7.4, 100 mm NaCl, and 0.1% Nonidet P-40. The beads were then boiled in 20 μl of 1X Laemmli SDS sample buffer (Bio-Rad). 6% of the sample inputs, 6% of the unbound fractions (where indicated), and the entire eluates from the immunoprecipitations were resolved on a 10% Tris gel (Bio-Rad) with Tris-Glycine SDS running buffer, transferred to a Immobilon PVDF membrane (EMD Millipore), and binding was monitored by radiometric analysis with a PharosFX Plus molecular imaging system (Bio-Rad).

##### Tandem Affinity Purification of Katanins

The LAP-KATNA1, KATNAL1, KATNAL2, KATNB1, and KATNBL1 inducible stable cell lines were grown in roller bottles and induced with .2 μg/ml Dox for 16 h in the presence of 100 nm Taxol prior to harvesting cells, as described in (39). Mitotic cells were then harvested in the presence of protease (Thermo Scientific), phosphatase (Thermo Scientific), and proteasome inhibitors (MG132, Enzo Lifesciences, Farmingdale, NY). LAP-KATNA1, KATNAL1, KATNAL2, KATNB1, and KATNBL1 were purified from cleared extracts using a previously established tandem affinity purification protocol (39).

##### In-gel Protein Digestions

Sample lanes from SDS-PAGE were sliced into six pieces and placed into individual micro-centrifuge tubes. Each gel slice was dehydrated with 100% acetonitrile for 30 min. Cysteines were reduced with 100 mm dithiothreitol in 50 mm ammonium bicarbonate for 60 min at 37^°^C, followed by the removal of buffer, and subsequently alkylated with 55 mm iodoacetamide in 50 mm ammonium bicarbonate for 45 min at room temperature in the dark. Buffer was decanted and gel slices were dehydrated with 100% acetonitrile followed by rehydration with 50 mm ammonium bicarbonate, repeated twice, except swelling in 5 ng/μl trypsin with the second 50 mm ammonium bicarbonate rehydration step on ice for 45 min. Trypsin solution was decanted and samples were incubated at 37^°^C overnight. Peptides were extracted from the gel slices using 100 μl of 50% acetonitrile for 20 min using water-bath sonication, repeated twice. Extracted peptides were dried using SpeedVac and reconstituted in 80 μl of 3% acetonitrile with 0.1% formic acid. Peptides were desalted using C18 StageTips as previously described (42).

##### Nano-liquid Chromatography with Tandem Mass Spectrometry (LC-MS/MS) Analysis

Nano-LC-MS/MS with collision-induced dissociation was performed on an Orbitrap XL (Thermo Scientific) integrated with an Eksigent 2D nano-LC instrument. A laser-pulled reverse-phase column, 75 μm × 200 mm, containing 5-μm C18 resin with 300-Å pores (AcuTech, Vista, CA) was used for online peptide chromatography. Electrospray ionization conditions using the nanospray source (Thermo Scientific) for the Orbitrap were set as follows: capillary temperature at 200 °C, tube lens at 110 V, and spray voltage at 2.3 kV. The flow rate for reverse-phase chromatography was 300 nl/min for loading and analytical separation (buffer A, 0.1% formic acid and 3% acetonitrile; buffer B, 0.1% formic acid and 100% acetonitrile). Peptides were loaded onto the column for 30 min and resolved by a gradient of 0–40% buffer B over 60 min. The Orbitrap was operated in data-dependent mode with a full precursor scan at high resolution (60,000 at *m*/*z* 400) from 300–1800 *m*/*z* and 10 MS/MS fragmentation scans at low resolution in the linear trap using charge-state screening excluding both unassigned and +1 charge ions. For collision-induced dissociation, the intensity threshold was set to 500 counts, and a collision energy of 40% was applied. Dynamic exclusion was set with a repeat count of 1 and exclusion duration of 30 s.

##### Experimental Design and Statistical Rational

**Fig. 1.**
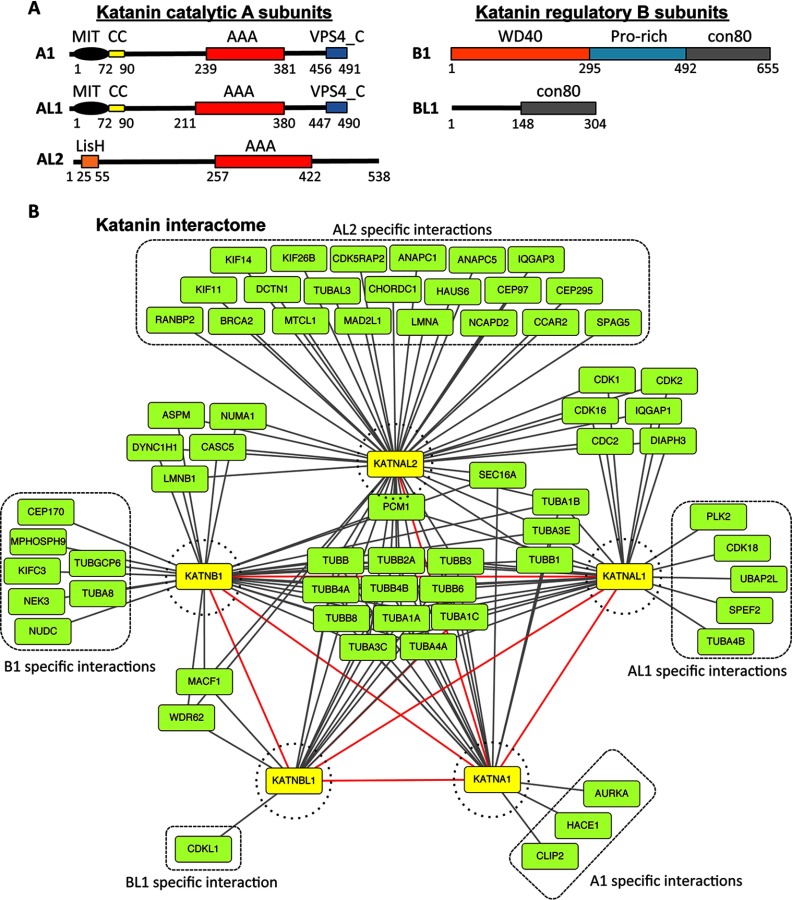
**The mammalian Katanin interactome (Katan-ome).** *A*, Domain architecture of the KATNA1 (UniProt ID O75449), KATNAL1 (UniProt ID Q9BW62), KATNAL2 (UniProt ID Q8IYT4), KATNB1 (UniProt ID Q9BVA0), and KATNBL1 (UniProt ID Q9H079) proteins. The number of amino acid residues are indicated for each protein. Major domains within each Katanin are annotated and include the microtubule interacting and trafficking domain (MIT), coiled coil domain (CC), AAA ATPase domain (AAA), VPS4 C-terminal domain (VPS4_C), proline-rich domain (Pro-rich) and the C-terminal region that binds to the N-terminal domain of KATNA1 (con80). *B*, The human Katanin interactome, Katan-ome. Cytoscape analysis of the KATNA1, KATNAL1, KATNAL2, KATNB1 and KATNBL1 interactomes assembled from the proteins identified by mass spectrometric analyses of each Katanin subunit purification from mitotic cells. Highlighted in yellow nodes are the Katanin bait proteins and green nodes represent the identified interacting proteins. Red edges represent interactions between Katanin subunits. Subunit specific interactions are highlighted with dashed boxes. See supplemental Tables S1 and S2 for a complete list of identified proteins and their associated information.

Database searches of the acquired spectra were analyzed with Mascot (v2.4; Matrix Science, Boston, MA). The UniProt human database (March 19, 2014; 88,647 sequences; 35,126,742 residues) was used. The following search parameters were used: trypsin digestion allowing up to two missed cleavages, carbamidomethyl on cysteine as a fixed modification, oxidation of methionine as a variable modification, 10-ppm peptide mass tolerance, and 0.5-Da fragment mass tolerance. With these parameters, an overall 5% peptide false discovery rate, which accounts for total false positives and false negatives, was obtained using the reverse UniProt human database as the decoy database. Peptides that surpassed an expectation cut-off score of 20 were accepted. All raw mass spectrometry files can be accessed at the UCSD Center for Computational Mass Spectrometry MassIVE data sets ftp://MSV000079358@massive.ucsd.edu (login: Torres, password: mitosis1). Peptides meeting the above criteria were filtered further. First, peptides corresponding to common contaminants identified in the CRAPome (43) and an internal LAP purification database were excluded from further analysis. The lists of proteins identified from each of the Katanin subunit purifications as a single pass are listed in supplemental Table S1 and the spectrums of significant single peptides are in supplemental Table S2. The final Katanin interactor lists were compiled and Cytoscape (44), an open access software used for complex network analysis and visualization, was used to generate the human Katanin interactome (Katan-ome) (Fig. 1*B*).

##### Immunofluorescence Microscopy

Immunofluorescence was carried out essentially as described previously (45) with minor modifications. HeLa stable cell lines were induced to express LAP-KATNA1, KATNAL1, KATNAL2, KATNB1, and KATNBL1 for 16 h, fixed with 4% paraformaldehyde, permeabilized with 0.2% Triton X-100/PBS, and costained with 0.5 μg/ml Hoechst 33342 and the indicated antibodies. Images were captured with a Leica (Buffalo Grove, IL) DMI6000 microscope (Leica DFC360 FX Camera, 63×/1.40–0.60 NA oil objective, Leica AF6000 software) at room temperature. Images were deconvolved with Leica Application Suite 3D Deconvolution software and exported as TIFF files.

##### Preparation of GOPTS-PEG-biotin Functional Coverslips

Coverslips (22 mm × 30 mm) were cleaned by incubating with 2% Hellmanex^®^ II solution at 60^°^C for 60 min, rinsed five times with Milli-Q water and dried in an oven. In the silanization step, a drop of (3-Glycidyloxypropyl)-trimetoxysilane (GOPTS) (Sigma, 440167) was sandwiched between every two coverslips and baked at 70^°^C for 60 min. Each coverslip was then rinsed through three beakers of 30 ml glass-distilled acetone (Electron Microscopy Sciences, Hatfield, PA, 10015) and air-dried. To functionalize coverslips with PEG-Biotin, 144 mg of methoxy PEG Amine MW 3000 (Jenkem Technology, Plano, TX) and 16 mg of biotin-PEG-NH_2_ MW 3400 (Laysan Bio, Arab, AL) were dissolved in 700 μl DMF and 35 μl was applied between each pair of GOPTS silanized coverslips followed by 75^°^C incubation in an oven for 8 h. The coverslip sandwiches were quickly disassembled, rinsed in Milli-Q water, air-dried and stored at 4^°^C.

##### Katanin Microtubule-severing TIRF Assays

Flow cells were constructed with GOPTS-PEG-biotin functional coverslips and glass slides were separated by double-sided tape, four chambers were made for each coverslip. To prepare for microtubule immobilization, each chamber was first incubated with blocking buffer (5% pluronic F-127, 100 μg/ml casein, 1× PBS) for 1 min, followed by 1 min incubation in 80 nm streptavidin in BRB80 (80 mm PIPES pH 7.5, 2 mm MgCl_2_, 2 mm EGTA). Chambers were rinsed with wash buffer (80 mm PIPES pH 7.5, 2 mm MgCl_2_, 2 mm EGTA, 40 mm d-glucose, 10 mm DTT) after each step. Microtubules labeled with 10% rhodamine and 10% biotin were added and allowed to bind for 3 min. Chambers were then rinsed with TIRF buffer (80 mm PIPES pH 7.5, 2 mm MgCl_2_, 2 mm EGTA, 40 mm d-glucose, 10 mm DTT, 5 mm ATP, 1 mg/ml glucose oxidase, 1 mg/ml catalase) to remove unattached microtubules. To record time-lapse assays, the flow cell was placed on a DMI6000 TIRF microscope (Leica) and microtubules were brought to focus. 20 μl of Katanin subunits were quickly flowed through the chamber at the indicated concentrations in TIRF buffer and images were collected every 10 s for 7 min. To measure the rate of microtubule severing, the average fluorescence intensities of the time-lapse images were measured using ImageJ and the background fluorescence was subtracted.

##### Recombinant Protein Expression and Purification

GST-fusion Katanin protein constructs were expressed in *Escherichia coli* BL21 (DE3) cells, grown in 6 L LB media with ampicillin (100 μg/ml) to an optical density of 0.6–0.8, and induced using 0.3 mm isopropyl thio-β-d-galactoside for ∼18 h at 16^°^C. Fresh cultures were harvested and resuspended in 100 ml lysis buffer (50 mm Tris-HCl pH 8.0, 300 mm NaCl, 2 mm MgCl_2_, 10% glycerol, 0.5 mm ATP, 1 mm DTT and complete EDTA-free protease inhibitor mixture (Roche, Basel, Switzerland)) followed by lysis with a microfluidizer® M110-P. Lysates were cleared by centrifugation at 15K RPM, 45 min, 4^°^C and incubated with Glutathione Agarose (Thermo Scientific) for 2 h at 4^°^C. Bound proteins were washed three times with wash buffer (50 mm Tris-HCl pH 8.0, 200 mm NaCl, 2 mm MgCl_2_, 10% glycerol, 0.5 mm ATP, 1 mm DTT) and eluted with 50 mm glutathione in storage buffer (20 mm HEPES pH 7.8, 50 mm KCl, 1 mm MgCl_2_, 10% glycerol, 0.25 mm ATP, 1 mm DTT), followed by dialysis into storage buffer at 4^°^C overnight. Dialyzed elutions were flash frozen in aliquots and stored at −80^°^C until use to prevent multiple freeze-thaw cycles. Katanin protein concentrations were determined using the Bradford reagent (Bio-Rad) and comparing to bovine serum albumin (BSA) standards with a NanoDrop 2000 (Thermo Scientific). For *in vitro* binding experiments, ^35^S-radiolabeled Katanins were expressed using the TnT® Quick Coupled Transcription/Translation System (Promega) (41).

##### Antibodies

Immunofluorescence, immunoblotting, and immunoprecipitations were carried out using the following antibodies: GFP (Abcam, Cambridge, UK), Gapdh (GeneTex, Irvine, CA), α-tubulin (Serotec, Raleigh, NC), HA (Cell Signaling, Danvers, MA). Secondary antibodies conjugated to FITC, Cy3 and Cy5 were from Jackson Immuno Research (West Grove, PA).

## RESULTS

### 

#### 

##### Defining the Mammalian Katanin Interactome (Katan-ome)

Our recent proteomic screen to identify novel mitotic microtubule co-purifying proteins led to the identification of KATNAL1 (NP_115492.1), a hypothetical KATNA1-like protein (38, 39). Interestingly, KATNA1, KATNB1, and a KATNB1-like protein 1 **(**C15orf29, KATNBL1) co-purified with KATNAL1, indicating that microtubule-severing was complex in higher eukaryotes and that potentially multiple Katanin heterodimers were involved in microtubule-severing (39). To better understand the human Katanins and more broadly the mechanisms controlling mammalian microtubule-severing, we sought to define the mammalian Katanin interactome. To do this, we generated doxycycline-inducible localization and affinity purification (LAP = EGFP-TEV-S-Peptide)-tagged-KATNA1, KATNAL1, KATNAL2, KATNB1, and KATNBL1 HeLa stable cell lines (39) (Fig. 1*A*). These cell lines were used to express and tandem affinity purify LAP-Katanins from cells arrested in mitosis with the treatment of Taxol. The purification eluates were resolved by SDS-PAGE and six gel slices containing the entire eluates were excised, trypsinized, and the interacting proteins were identified by 2D-LC MS/MS (supplemental Tables S1 and S2). The mass spectrometry data from each purification was used to generate an interaction module for each subunit and the Katanin interactome (Katan-ome) was assembled from the five individual interaction modules using Cytoscape (44) (Fig. 1*B*). Several insights were gained from the Katan-ome. First, although KATNA1, KATNAL1, KATNB1, and KATNBL1 were able to copurify with each other in reciprocal purifications, KATNAL2 only co-purified with KATNA1 (Fig. 1*B* and supplemental Tables S1 and S2). Second, each Katanin interacted with a unique subset of proteins composed primarily of spindle-associated proteins. Third, all Katanins shared interactions with microtubule-associated proteins and finally, all Katanins interacted with various tubulin isoforms (Fig. 1*B* and supplemental Tables S1 and S2).

##### Validation of Katanin Subunit Interactions

**Fig. 2.**
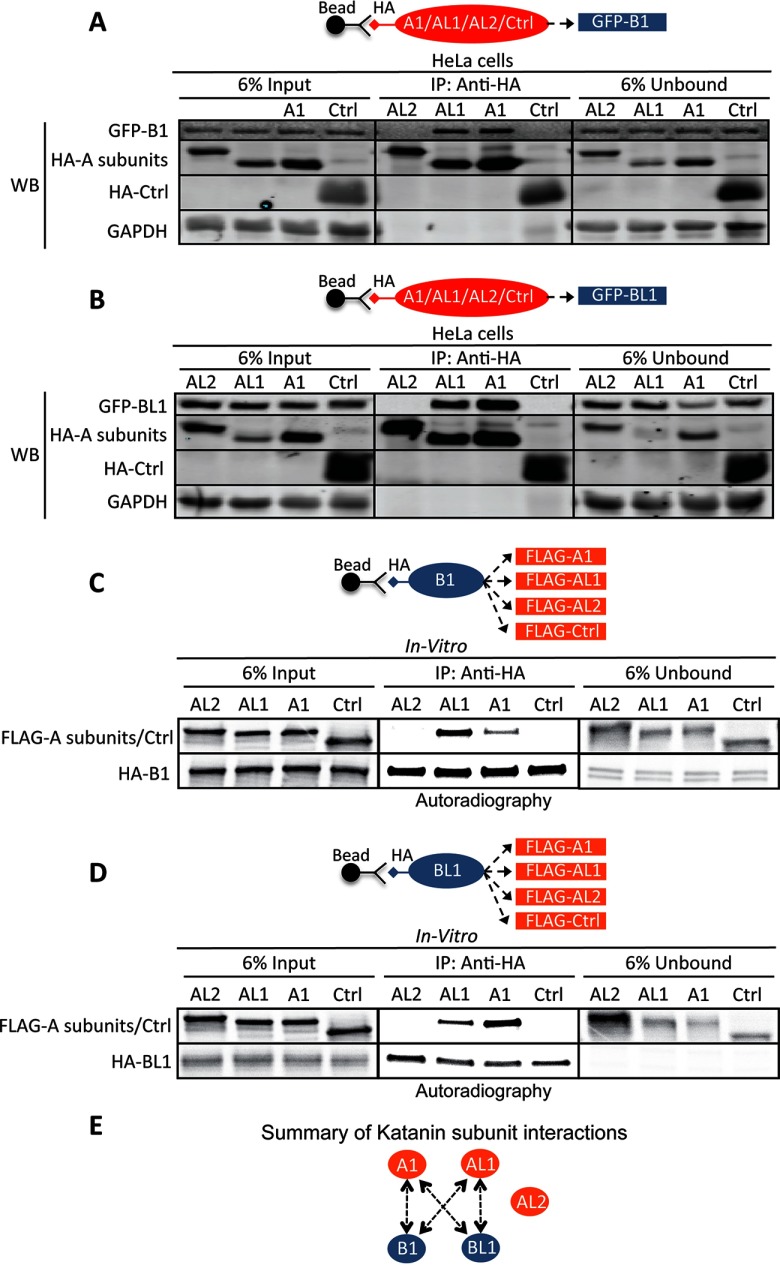
**The Katanin-Katanin pairwise binding assays.** *A, B*, In cell Katanin subunit pairwise binding reactions. LAP-tagged GFP-B1 (*A*) or GFP-BL1 (*B*) HeLa stable cell lines were transfected with HA-tagged A1, AL1, or AL2 subunits or the negative control (Ctrl) STARD9-START. HA-A1, HA-AL1, HA-AL2, or HA-Ctrl were immunoprecipitated from 140 μg of protein extracts. Six percent of the input and all of the immunoprecipitates were western blotted (WB) for the indicated GFP-tagged B subunits (using anti-GFP antibodies) and the HA-tagged A subunits or HA-Ctrl (using anti-HA antibodies). Note that the negative control HA-Ctrl does not bind to either Katanin B subunit. Additionally, the negative control GAPDH protein is not found in any of the immunoprecipitations. *C, D*, *In vitro* ^35^S-radiolabeled Katanin subunit pairwise binding reactions. *In vitro* transcribed and translated ^35^S-radiolabeled FLAG-tagged A subunits or negative control (Ctrl) STARD9-MD and HA-tagged B1 (*C*) or BL1 (*D*) subunits were used for pairwise *in vitro* binding reactions as indicated. HA-tagged Katanin B subunits were then immunoprecipitated and the radiolabeled Katanin A and B subunits and Ctrl in the immunoprecipitates were visualized by autoradiography. Note that FLAG-A1 and FLAG-AL1 co-precipitate with HA-B1 and HA-BL1, whereas FLAG-AL2 and the negative control FLAG-Ctrl do not. *E*, Summary of in cell and *in vitro* binding data. Arrows indicate the direction of the interaction, double arrows indicate that the interaction was recapitulated in reciprocal co-immunoprecipitations. See supplemental Fig. S1 for in cell and *in vitro* reciprocal Katanin subunit binding data.

To further validate the interactions between the Katanin subunits and to determine if these interactions were direct, we performed pairwise binding assays from cell extracts and an *in vitro* system in the presence of the microtubule depolymerizing agent nocodazole. First, LAP-Katanin B subunit expressing cell lines were transfected with HA-tagged Katanin A subunits and the A subunits were immunoprecipitated and the immunoprecipitates were immunoblotted for LAP-Katanin B subunits (Fig. 2*A* and 2*B*). Similar reciprocal co-IPs were carried out with LAP-Katanin A subunit expressing cell lines that had been transfected with HA-tagged Katanin B subunits (supplemental Fig. S1*A–*S1*B*). Finally, reciprocal co-IPs were carried out with LAP-Katanin B subunit expressing cell lines that had been transfected with HA-tagged Katanin B subunits (supplemental Fig. S1*C–*S1*D*). This subunit binding analysis revealed that KATNB1 and KATNBL1 were each able to associate with KATNA1 and KATNAL1 but not with KATNAL2. Additionally, no interaction was detected between KATNB1 and KATNBL1. Next we sought to determine whether the observed interactions between KATNB1 and KATNBL1 with KATNA1 and KATNAL1 were direct. To do this, we performed pairwise binding *in vitro* co-IPs with *in vitro* transcribed and translated S35 labeled A and B subunits. Consistent with our cell extract binding assays both KATNB1 and KATNBL1 bound to KATNA1 and KATNAL1 directly (Fig. 2*C* and 2*D* and supplemental Fig. S1*E*). Together these data indicated that KATNB1 and KATNBL1 could each bind to either KATNA1 or KATNAL1 directly and that KATNAL2 failed to interact with any of the Katanin subunits (Fig. 2*E*).

##### KATNBL1 has a Dynamic Cell Cycle Dependent Subcellular Localization

**Fig. 3.**
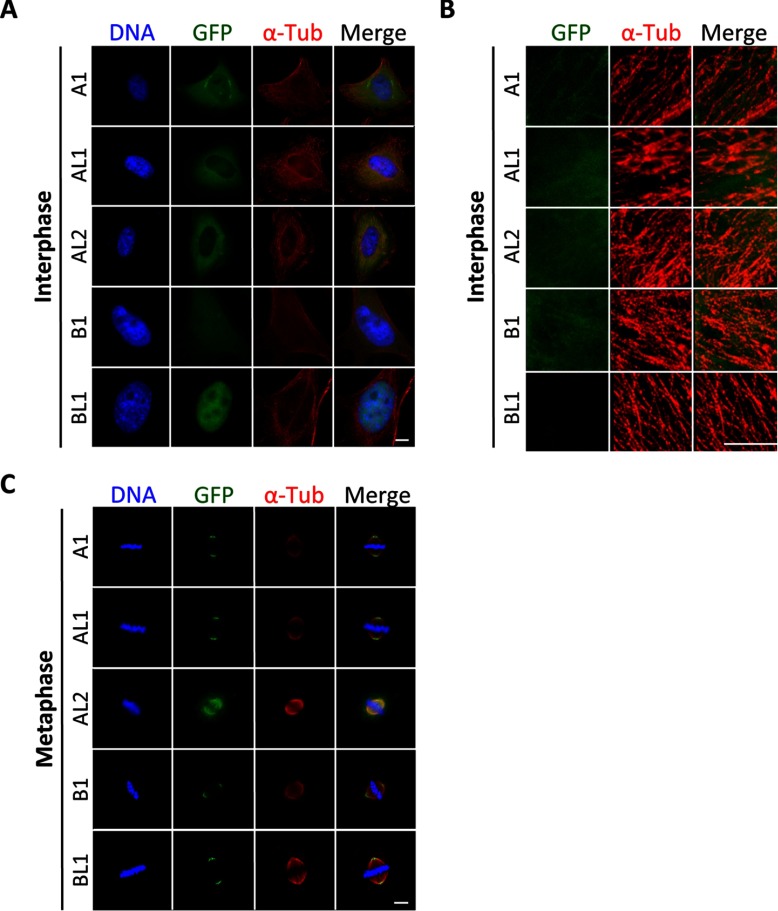
**Katanin subunit cell cycle subcellular localization.** *A–C*, Immunofluorescence microscopy of paraformaldehyde fixed LAP-tagged GFP-Katanin subunit expressing HeLa cells stained with Hoechst 33342 to detect the DNA, anti-α-tubulin antibodies to detect microtubules, and anti-GFP antibodies to detect GFP-Katanin subunit localization. Images show the subcellular localization of each Katanin subunit during interphase, either whole view (*A*) or zoomed view of cytoplasmic microtubules (*B*), and metaphase of mitosis (*C*). Note that KATNA1, KATNAL1, KATNAL2 and KATNB1 localize to the cytoplasm in interphase and the spindle poles during mitosis, whereas KATNBL1 localizes to the nucleus in interphase and spindle poles during mitosis. Bar = 5 μm.

Because KATNBL1 bound directly to KATNA1 and KATNAL1, we sought to determine if it also localized to the cytoplasm in interphase and the spindle poles during mitosis, as previously shown for KATNB1 (8). Immunofluorescence microscopy of HeLa cells expressing LAP-Katanin A or B subunits showed that KATNA1, KATNAL1, KANTAL2, and KATNB1 localized within the cytoplasm, partially overlapping with microtubules, in interphase and to the mitotic spindle and spindle poles during mitosis, consistent with previous reports (8, 35, 39, 46, 47) (Fig. 3*A–*3*C*). Surprisingly, KATNBL1 failed to localize to the cytoplasm in interphase cells and instead was enriched in the nucleus (Fig. 3*A* and 3*B*). However, similar to KATNA1, KATNAL1, KATNAL2, and KATNB1 it also localized to the spindle poles in mitosis (Fig. 3*C*). Together, these results indicated that compared with other Katanin A or B subunits, KATNBL1 was uniquely localized to the nucleus during interphase and only associated with spindle poles during mitosis.

##### Regulation of Katanin Microtubule-severing Activity by KATNBL1

**Fig. 4.**
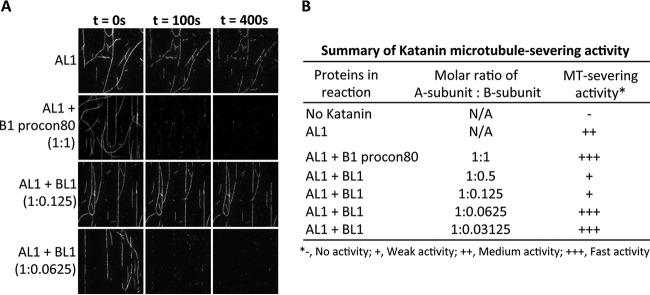
**KATNBL1 regulates Katanin microtubule-severing activity.** *A*, Total internal reflection fluorescence microscopy (TIRFM) *in vitro* microtubule-severing assays using recombinant purified GST-KATNAL1, rhodamine-labeled microtubules and the indicated GST-tagged B subunits. Time (t) is in seconds. *B*, Summary of the relative Katanin microtubule-severing activity (- = no activity, + = weak activity, ++ = medium activity, and +++ = fast activity) for the indicated molar ratio of A-subunit : B-subunit. Average fluorescence signals of time-lapse TIRF images were measured with ImageJ. Data represent results from three independent experiments. See supplemental Fig. S3 for quantification of time-lapse microtubule TIRFM fluorescence signals.

Although KATNA1 and KATNAL1 had been shown to have microtubule-severing activities (2, 8, 35), there had been no characterization of the putative KATNAL2 microtubule-severing activities. Therefore, we overexpressed KATNAL2 and analyzed its ability to sever microtubules by immunofluorescence microscopy. In contrast to cells overexpressing KATNA1 or KATNAL1 that displayed a loss of α–tubulin signal indicative of microtubule-severing, overexpression of KATNAL2 had no effect on the microtubule lattice, indicating that it had no detectable microtubule-severing activity (supplemental Fig. S2). Similarly, although KATNB1 had been shown to regulate microtubule-severing activity of KATNA1 (8), the effect of KATNBL1 on A subunit microtubule-severing had not been determined. Thus, we analyzed the effect of KATNBL1 on the KATNAL1 microtubule-severing activity using *in vitro* microtubule-severing assays coupled to live total internal reflection fluorescence microscopy (TIRFM). Briefly, recombinant KATNAL1 and KATNBL1 subunits were added to immobilized rhodamine labeled microtubules at various ratios and microtubule severing was monitored every 10 s for 7 mins using TIRFM. Consistent with previous results, KATNAL1 showed microtubule-severing activity, and this activity was enhanced by the addition of the KATNB1 procon80 domain at a 1:1 ratio (8) (Fig. 4*A* and 4*B* and supplemental Fig. S3). Interestingly the addition of full-length KATNBL1 at a 1:0.125 ratio (KATNAL1/KATNBL1) inhibited the microtubule-severing activity of KATNAL1 and increasing the concentration of KATNBL1 to a 1:0.5 ratio did not further inhibit KATNAL1 activity (Fig. 4*A* and 4*B* and supplemental Fig. S3). However, the addition of KATNBL1 at a ratio of 1:0.0625 enhanced KATNAL1 microtubule-severing activity and decreasing the concentration of KATNBL1 further to a ratio of 1:0.03125 did not further enhance the KATNAL1 activity (Fig. 4*A* and 4*B* and supplemental Fig. S3). Together, these results indicated that KATNBL1 was able to regulate the KATNAL1 microtubule-severing activity in a concentration dependent manner.

##### Sequestration of KATNBL1 to the Nucleus is Dependent on a Nuclear Localization Signal

**Fig. 5.**
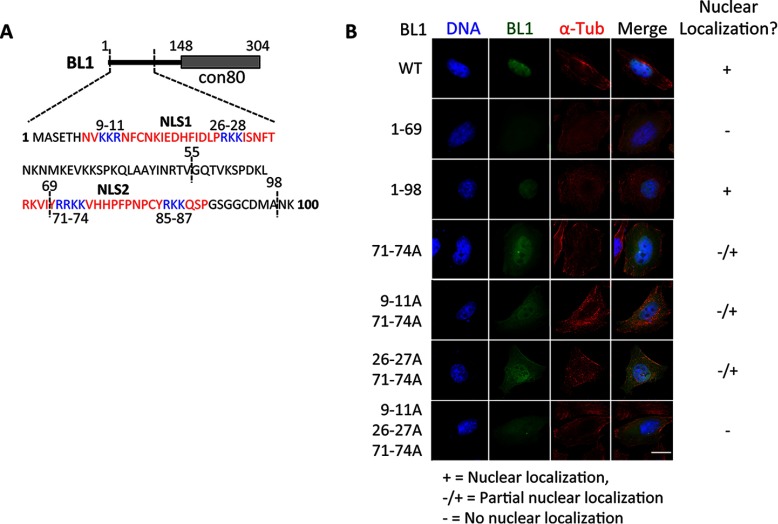
**KATNBL1 localization to the nucleus during interphase requires an N-terminal nuclear localization sequence.** *A*, cNLS Mapper analysis of KATNBL1 identified two putative bipartite nuclear localization signals (NLS) corresponding to amino acids 9–28 (NLS1) and 71–87 (NLS2). The generated KATNBL1 NLS1 and NLS2 mutants and KATNBL1 truncation mutants are indicated. *B*, Immunofluorescence microscopy, as in Fig. 3, was used to visualize the subcellular localization of KATNBL1 wildtype (WT), NLS1 mutants, NLS2 mutants, deletion mutants and N-terminal fusions as indicated. + indicates nuclear localization, ± indicates partial nuclear localization and - indicates no nuclear localization. Bar = 5 μm. Note that wildtype KATNBL1 localizes to the nucleus, whereas mutation of NLS2 basic cluster 1 (amino acids 71–74) in combination with NLS1 basic cluster 1 (amino acids 9–11) and 2 (amino acids 26–28) inhibits its transport to the nucleus and it remains in the cytoplasm. See supplemental Fig. S4 for the localization of additional mutants. For a list of primers used for generating KATNBL1 mutants see supplemental Table S3.

The strong localization of KATNBL1 to the nucleus during interphase indicated that KATNBL1 was being actively localized to the nucleus and could potentially harbor a nuclear localization signal (NLS). Analysis of the KATNBL1 amino acid sequence with cNLS Mapper (48) indicated that KATNBL1 had at least two putative bipartite NLS's; NLS1 corresponding to amino acids 9–28 (basic cluster 1 amino acids 9–11 and basic cluster 2 amino acids 26–28) and NLS2 corresponding to amino acids 71–87 (basic cluster 1 amino acids 71–74 and basic cluster 2 amino acids 85–87) (Fig. 5*A*). Therefore, we performed an analysis of NLS1 and NLS2 mutants, by substituting amino acid residues within these motifs to alanines and assessing their subcellular localization with immunofluorescence microscopy. Mutation of the NLS1 basic cluster 1 or basic cluster 1 and 2 had no effect on the localization of KATNBL1 and it remained in the nucleus (Fig. 5*A* and 5*B*). Consistently, a KATNBL1 truncation mutant missing the first 55 amino acids (Δ1–55) also maintained its nuclear localization and fusion of the first 69 N-terminal amino acids of KATNBL1 to GFP did not localize it to the nucleus (Fig. 5*A* and 5*B* and supplemental Fig. S4). This indicated that NLS1 was not necessary for KATNBL1 nuclear localization. Next, we mutated NLS2 basic cluster 1 or basic cluster 1 and 2. Interestingly, mutation of the NLS2 basic cluster 1 led to the redistribution of KATNBL1 from the nucleus to both the nucleus and cytoplasm, whereas mutation of both basic cluster 1 and 2 did not further redistribute KATNBL1 to the cytoplasm (Fig. 5*A* and 5*B* and supplemental Fig. S4). This indicated that NLS2 basic cluster 1 was important for the localization of KATNBL1 to the nucleus. Consistently, fusion of the first 98 N-terminal amino acids of KATNBL1 to GFP localized it to the nucleus (Fig. 5*A* and 5*B*). Furthermore, mutation of NSL1 basic cluster 1 or 2 in combination with NLS2 basic cluster 1 did not further redistribute KATNBL1 to the cytoplasm (Fig. 5*A* and 5*B*). However, mutation of all three (NLS1 basic cluster 1 and 2 and NLS2 basic cluster 1) led to the complete redistribution of KATNBL1 to the cytoplasm (Fig. 5*A* and 5*B*). Together, these data indicated that NLS2 basic cluster 1 was important for targeting KATNBL1 to the nucleus during interphase and that NLS1 basic cluster 1 and 2 also played a role.

##### KATNB1 Competes with KATNBL1 for Binding to KATNA1 and KATNAL1

**Fig. 6.**
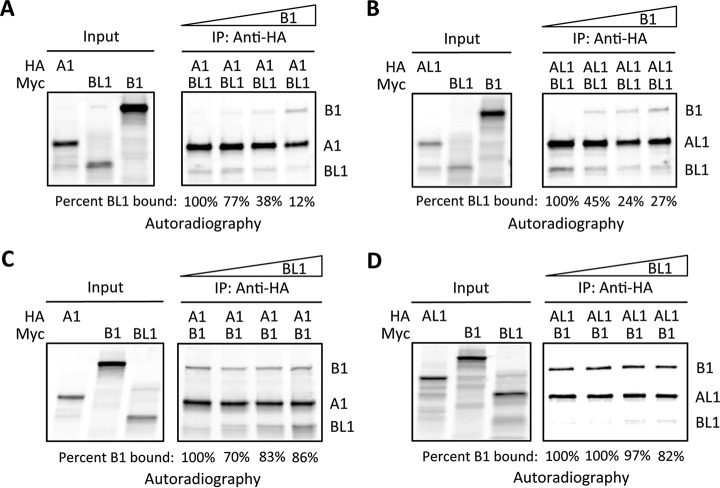
**KATNB1 competes the KATNA1-KATNBL1 and KATNAL1-KATNBL1 interactions.** *A–D*, Competition binding assays, using increasing concentrations of recombinant KATNB1 or KATNBL1. HA or Myc-tagged *in vitro* ^35^S-radiolabeled Katanin subunits were used in pairwise binding competition reactions as indicated and the results of the competition binding assays were visualized by autoradiography. *A, B*, Increasing concentrations of KATNB1 competes the KATNA1-KATNBL1 and KATNAL1-KATNBL1 interactions. The intensity of the KATNBL1 bands were measured using ImageJ and the percent KATNBL1 bound is indicated. Note that the percent KATNBL1 bound decreases as the concentration of KATNB1 increase. *C–D*, Increasing concentrations of KATNBL1 weakly compete the KATNA1-KATNB1 and KATNAL1-KATNB1 interactions. The intensity of the KATNB1 bands were measured using ImageJ and the percent KATNB1 bound is indicated. Note that the percent KATNB1 bound decreases only slightly as the concentration of KATNBL1 increases.

Because KATNB1 and KATNBL1 both interacted directly with KATNA1 and KATNAL1 it was possible that they were competing for binding to KATNA1 and KATNAL1. To test this, we performed *in vitro* Katanin A and B subunit binding experiments as described previously, except that increasing concentrations of recombinant B subunits were added. Indeed, KATNB1 was able to compete the KATNA1-KATNBL1 and KATNAL1-KATNBL1 interactions in a dose dependent manner (percent KATNBL1 bound decreased from 100% to ∼20%) (Fig. 6*A* and 6*B*). However, reciprocal competition studies with increasing concentrations of KATNBL1 showed that KATNBL1 was only able to weakly compete the KATNA1-KATNB1 and KATNAL1-KATNB1 interactions (percent KATNB1 bound decreased from 100% to ∼84%) (Fig. 6*C* and 6*D*). Together, these results indicated that KATNB1 and KATNBL1 were likely in competition for binding to KATNA1 and KATNAL1 and that KATNB1 had a higher affinity for KATNA1 and KATNAL1 than KATNBL1.

## DISCUSSION

By coupling Katanin subunit tandem affinity purifications from mitotic cells with mass proteomic analyses, we identified the protein interaction module for each of the five human Katanin subunits. These interaction modules were combined to generate the human Katanin interactome (Katan-ome): a mitotic protein network comprised of all the Katanin subunits and their interacting partners (Fig. 1*B*). The identified protein-protein interactions could be used to further understand the cells microtubule-severing machinery, their mechanisms of action and their regulation. For example, the Katanin interactors may represent factors that regulate Katanin activity, localization, complex assembly or that coordinate with the Katanins to promote microtubule-severing and the generation of microtubule density that is required for proper spindle assembly and cell division. Along these lines, reports in *Xenopus laevis* have indicated that Katanin function may be regulated through posttranslational modifications like phosphorylation (49–51). Interestingly several kinases were identified in the Katan-ome, including Aurora A (KATNA1 interactor); PLK2 (KANTAL1 interactor); CDK1 and CDK2 (KATNAL1 and KATNAL2 interactors); and the kinase regulatory protein CDK5RAP2 (KATNAL2 interactor) (Fig. 1*B* and supplemental Tables S1 and S2). Aurora A, PLK2, CDK1, CDK2, and CDK5RAP2 all have critical roles in mitosis predominantly through the regulation of centriole and centrosome homeostasis and could be modulating Katanin activity at the spindle poles during mitosis through phosphorylation (52–57). Interestingly, the Katan-ome contained a good degree of connectivity between the individual Katanin subunits, indicating that they may share common modes of inhibition/activation and/or may have redundant roles. However, our analysis of mitotically associated Katanin interacting proteins could have precluded the identification of important interactors that only associate with Katanins during interphase. Therefore, additional proteomic analyses of Katanin purifications from interphase cells could elucidate additional interacting proteins important for regulating microtubule-severing.

Our binding experiments indicated that KATNAL2 did not bind to other Katanin subunits, however it is possible that the epitope-tagged KATNAL2 was not properly folded or that different KATNAL2 isoforms were binding to themselves and potentially excluding other interactions, as has been shown for the five alternatively spliced mouse KATNAL2 isoforms (47). However, there is no evidence for alternatively spliced forms of KATNAL2 in humans. Additionally, KATNAL2 had more mitotic interactors than any other Katanin subunit in the network. KATNAL2 interactors included CDK5RAP2, and CEP295, which are important regulators of centriole amplification (52, 58); CDK5RAP2, WDR62, and CEP97, which are critical for maintaining a bipolar spindle and CDK5RAP2 also plays a role in promoting cytokinesis and maintaining a normal nuclear size (52, 59, 60); and CEP97 and PCM1, which have roles in primary cilium formation (59, 61). This is interesting in light of a recent study, which showed that mouse KATNAL2 has multiple roles in microtubule-based processes such as centriole amplification, spindle bipolarity, cytokinesis, nuclear morphology, and ciliogenesis (47). Although we were unable to detect KATNAL2 microtubule-severing activity in cells (supplemental Fig. S2), it is possible that KATNAL2 can regulate these cellular processes through its protein-protein interactions. Along these lines, in *Caenorhabditis elegans* the spindle assembly function of the Katanin A subunit MEI-1 does not require its microtubule-severing activity, indicating that Katanins may have microtubule-severing independent roles in spindle assembly (62).

A key unanswered question is how does KATNBL1 localize to the spindle poles during mitosis. KATNB1 has been shown to localize to the spindle poles through its WD40 domain, however KATNBL1 lacks the WD40 domain (7). Interestingly, KATNBL1 interacted with WDR62, which is known to localize to the spindle poles and is required for spindle organization (63). Therefore, it is possible that WDR62 may be targeting KATNBL1 to the spindle poles during mitosis and should be explored further. Additionally, KATNBL1 contains the C-terminal con80 domain that in KATNB1 binds to KATNA1 and our binding studies indicated that KATNBL1 was able to interact with KATNA1 and KATNAL1 (8, 62) (Fig. 2). Thus it is also possible that KATNBL1 localizes to the spindle poles through its interaction with KATNA1 and KATNAL1. Whereas KATNB1 was able to robustly compete the KATNA1-KATNBL1 and KATNAL1-KATNBL1 interactions in a dose dependent manner (Fig. 6*A* and 6*B*), KATNBL1 was only able to weakly compete the KATNA1-KATNB1 and KATNAL1-KATNB1 interactions (Fig. 6*C* and 6*D*), indicating that *in vitro* KATNBL1 had a weaker association with KATNA1 and KATNAL1 compared with KATNB1. Interestingly, these observations are consistent with a previous study showing that the N terminus of KATNAL1 bound to either KATNB1 or KATNBL1 and that depletion of the first 29 amino acids of KATNAL1 drastically decreased its association with KATNBL1 and only had a minor effect on its association with KATNB1 (62). These data indicate that KATNB1 likely has multiple sites of interaction with KATNAL1 and therefore we would expect KATNB1 to have a stronger association with KATNAL1 compared with KATNBL1. Finally, the inhibition of KATNAL1 microtubule-severing activity with equimolar concentrations of KATNBL1 was surprising (Fig. 4*A* and 4*B*), however a possible explanation could be that each KATNBL1 monomer binds to a KATNAL1 monomer and associates it with microtubules, thereby depleting the levels of free KATNAL1 monomers that are available to form homo-hexamers needed for microtubule-severing. Consistent with this, the con80 domain that is found at the C terminus of KATNBL1 has been shown to bind to both microtubules and to the Katanin A subunit (8). Therefore, lower concentrations of KATNBL1 would facilitate KATNAL1 microtubule-binding and still allow the formation of KATNAL1 homo-hexamers, thereby stimulating microtubule-severing.

To our knowledge this is the first characterization of KATNBL1's function in regulating microtubule-severing. KATNBL1 co-purified and co-immunoprecipitated with KATNA1 and KATNAL1 indicating that it was associating directly with these enzymes in cells (Figs. 1*B* and 2). *In vitro*, KATNBL1 was able to regulate the microtubule-severing activity of KATNAL1 in a concentration dependent manner (Fig. 4 and supplemental Fig. S3). However, the localization of KATNBL1 to the nucleus during interphase implies that KATNBL1 is only able to bind and regulate KATNA1 and KATNAL1 microtubule-severing during mitosis, when the nuclear membrane is dissolved. This also implies that KATNB1 and KATNBL1 may be in competition/equilibrium to stimulate or inhibit Katanin microtubule-severing during mitosis. How KATNB1 and KATNBL1 coordinate and are regulated to ensure the appropriate amount of microtubule-severing during mitosis remains to be explored.

## Supplementary Material

Supplemental Data

## Footnotes

^1^ The abbreviations used are:

MIT: microtubule interacting and trafficking domain.
