# Supplementary material for: Proteomic Analysis of the Mammalian Katanin Family of Microtubule-severing Enzymes Defines Katanin p80 subunit B-like 1 (KATNBL1) as a Regulator of Mammalian Katanin Microtubule-severing
Source: Mol Cell Proteomics. 2016 Feb 29;15(5):1658–69. doi: 10.1074/mcp.M115.056465 (PMC4858946; doi:10.1074/mcp.M115.056465)
Supplement: Supplemental Data [file supp_15_5_1658__index.html]

Proteomic Analysis of the Mammalian Katanin Family of Microtubule-severing Enzymes Defines KATNBL1 as a Regulator of Mammalian Katanin Microtubule-severing — Proteomic Analysis of the Mammalian Katanin Family of Microtubule-severing Enzymes Defines Katanin p80 subunit B-like 1 (KATNBL1) as a Regulator of Mammalian Katanin Microtubule-severing — KATNBL1 is a Regulator of Microtubule-severing — Supplemental Data 

# Proteomic Analysis of the Mammalian Katanin Family of Microtubule-severing Enzymes Defines Katanin p80 subunit B-like 1 (KATNBL1) as a Regulator of Mammalian Katanin Microtubule-severing

## Supplemental Data

- Supplemental Material (.pdf, 4.3 MB) - Supplemental Material
- Supplemental Table S1 (.xlsx, 62 KB) - Supplemental Table S1
- Supplemental Table S2 (.xlsx, 318 KB) - Supplemental Table S2
- Supplemental Table S3 (.docx, 192 KB) - Supplemental Table S3
