# Supplementary material for: Proteomic Analysis of the Mammalian Katanin Family of Microtubule-severing Enzymes Defines Katanin p80 subunit B-like 1 (KATNBL1) as a Regulator of Mammalian Katanin Microtubule-severing
Source: Mol Cell Proteomics. 2016 Feb 29;15(5):1658–69. doi: 10.1074/mcp.M115.056465 (PMC4858946; doi:10.1074/mcp.M115.056465)
Supplement: Supplemental Data [file 10.1074_M115.056465_mcp.M115.056465-1.pdf]

SUPPLEMENTAL MATERIAL

Proteomic Analysis of the Mammalian Katanin Family of Microtubule-severing Enzymes Defines KATNBL1 as a Regulator of Mammalian Katanin Microtubule-severing

Keith Cheung[1], Silvia Senese[1], Jiaen Kuang[1], Ngoc Bui[1], Chayanid Ongpipattanakul[1], Ankur Gholkar[1], Whitaker Cohn[2], Joseph Capri[2], Julian P. Whitelegge[2,3,4] and Jorge Z. Torres[1,3,4*]

[1]Department of Chemistry and Biochemistry, University of California, Los Angeles, CA 90095, USA
[2]Pasarow Mass Spectrometry Laboratory, The Jane and Terry Semel Institute for Neuroscience and Human Behavior, David Geffen School of Medicine, University of California, Los Angeles, CA 90095, USA
[3]Molecular Biology Institute, University of California, Los Angeles, CA 90095, USA
[4]Jonsson Comprehensive Cancer Center, University of California, Los Angeles, CA 90095, USA

*Corresponding author:

Jorge Z. Torres
607 Charles E. Young Drive East
Los Angeles, CA 90095
Phone: 310-206-2092
Fax: 310-206-5213
torres@chem.ucla.edu

Supplemental Figures

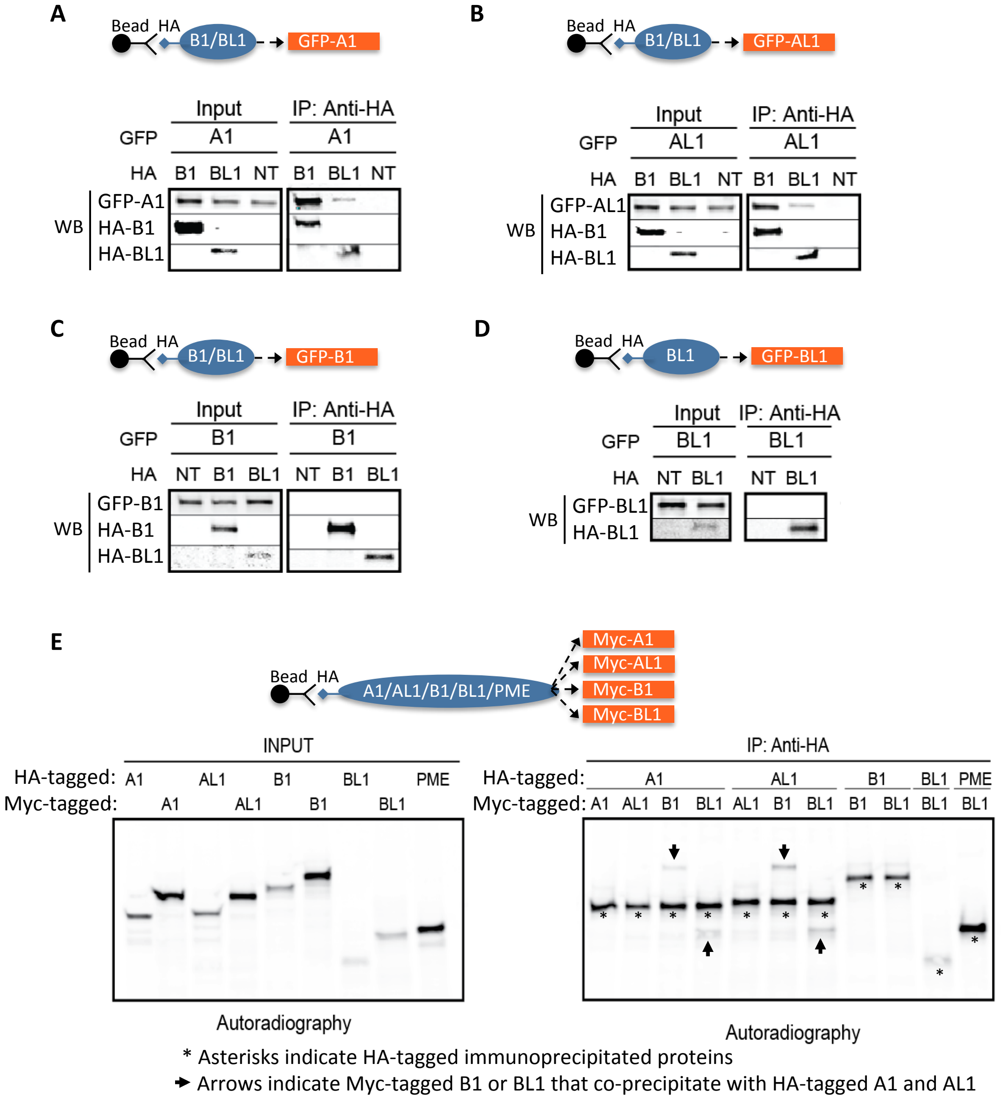

Supplemental Fig. S1. **Katanin subunit interactions.** (*A-D*) In cell Katanin subunit pairwise binding reactions. (*A-B*) LAP-tagged GFP-A1 (*A*) or GFP-AL1 (*B*) HeLa stable cell lines were transfected with HA-tagged B1 or BL1 subunits. HA-B1 or HA-BL1 were immunoprecipitated from 140μg of protein extracts. 6% of the input and all of the immunoprecipitates were western blotted (WB) for the indicated GFP-tagged A subunits (using anti-GFP antibodies) and the HA-tagged B subunits (using anti-HA antibodies). NT denotes non-transfected. (*C-D*) same as in *A-B*, except that LAP-tagged GFP-B1 (*C*) or GFP-BL1 (*D*) HeLa stable cell lines were transfected with HA-tagged B1 or BL1 subunits. (*E*) *In vitro* 35S-radiolabeled Katanin subunit pairwise binding reactions. *In vitro* transcribed and translated 35S-radiolabeled HA-tagged A subunits or B subunits or Myc-tagged A subunits or B subunits were used for pairwise *in vitro* binding reactions as indicated. HA-tagged Katanin subunits were then immunoprecipitated and the radiolabeled Katanin subunits in the immunoprecipitates were visualized by autoradiography. The bands corresponding to the immunoprecipitated HA-tagged Katanin subunits contain an asterisk below them. PME denotes the negative control protein PME-1. Note that Myc-B1 and Myc-BL1 (indicated by arrows) co-precipitate with both HA-A1 and HA-AL1.

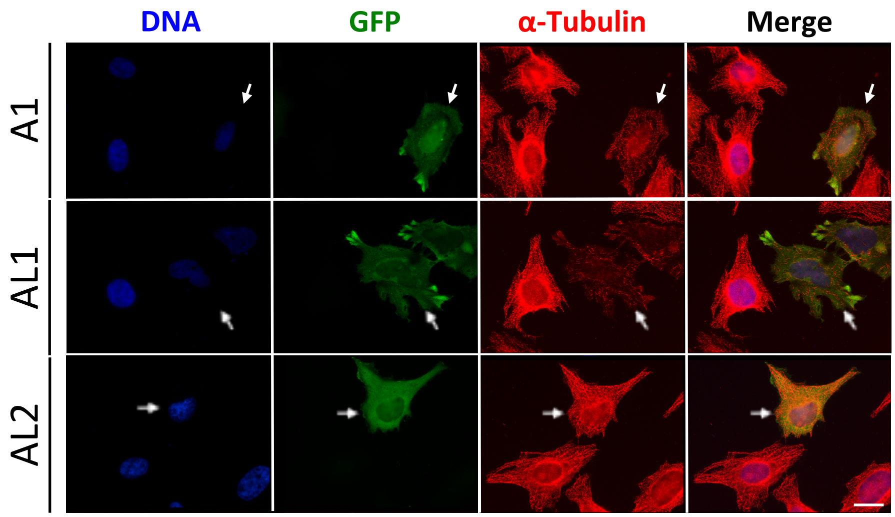

Supplemental Fig. S2. **KATNAL2 does not display microtubule-severing activity.** Immunofluorescence microscopy of cells transfected with GFP-KATNA1, GFP-KATNAL1 or GFP-KATNAL2 for 48 hours. Cells were fixed with paraformaldehyde and stained with Hoechst 33342 to detect the DNA, anti-α-tubulin antibodies to detect microtubules, and anti-GFP antibodies to detect GFP-Katanin subunit localization. Note that in cells overexpressing GFP-KATNA1 or GFP-KATNAL1 (see arrows) microtubules are severed, whereas microtubules remain stabilized in cells overexpressing GFP-KATNAL2. Bar= 5μm.

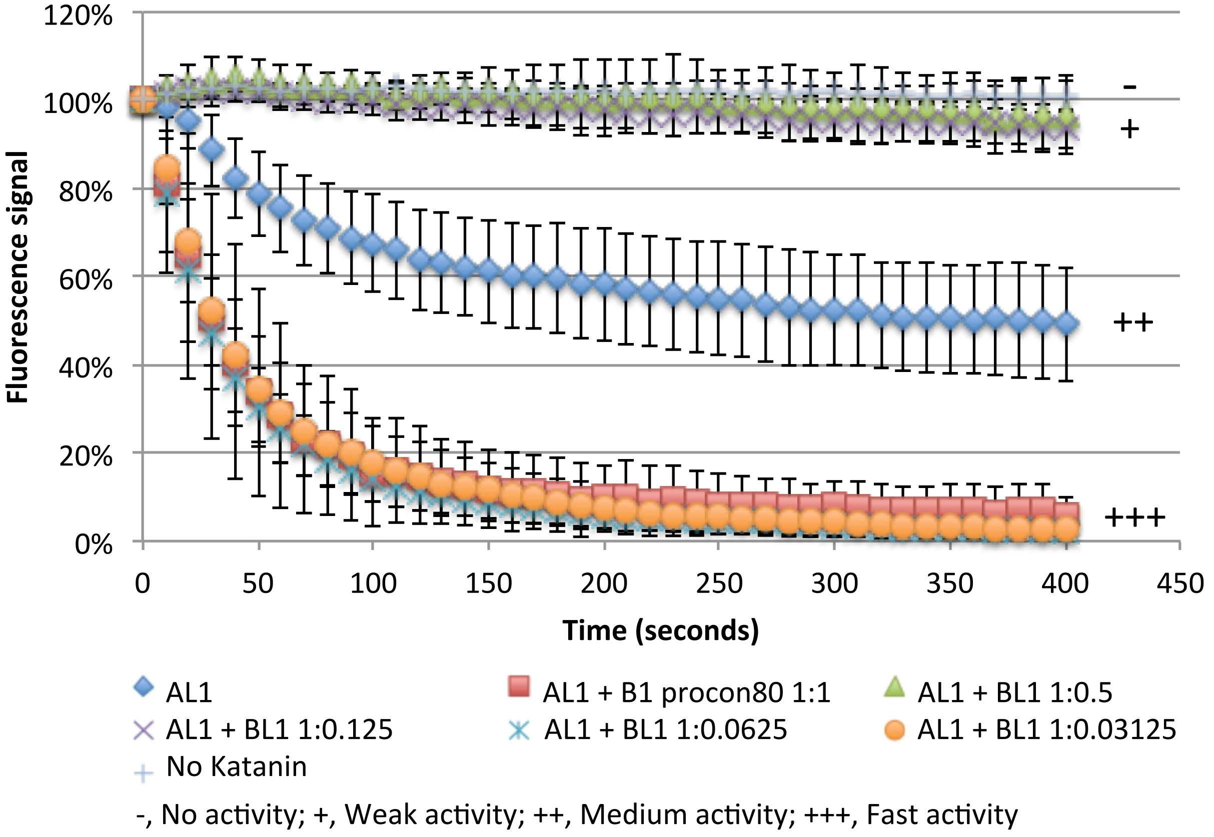

Supplemental Fig. S3. **Quantification of time-lapse microtubule TIRF fluorescence signals in the presence (or absence) of various katanin subunits.** Quantification of microtubule fluorescence signals of the TIRF data presented in Fig. 4B. Each data point represents the average of three individual experiments. Error bars indicate +/- SD. Average fluorescence signals of time-lapse TIRF images were measured with ImageJ. Each time point represents the percentage of the average fluorescence signal remaining in comparison to the initial amount of fluorescence signal at t = 0s. (+++) indicates fast microtubule-severing activity, (++) indicates medium microtubule-severing activity, (+) indicates weak microtubule-severing activity, and (-) no microtubule-severing activity.

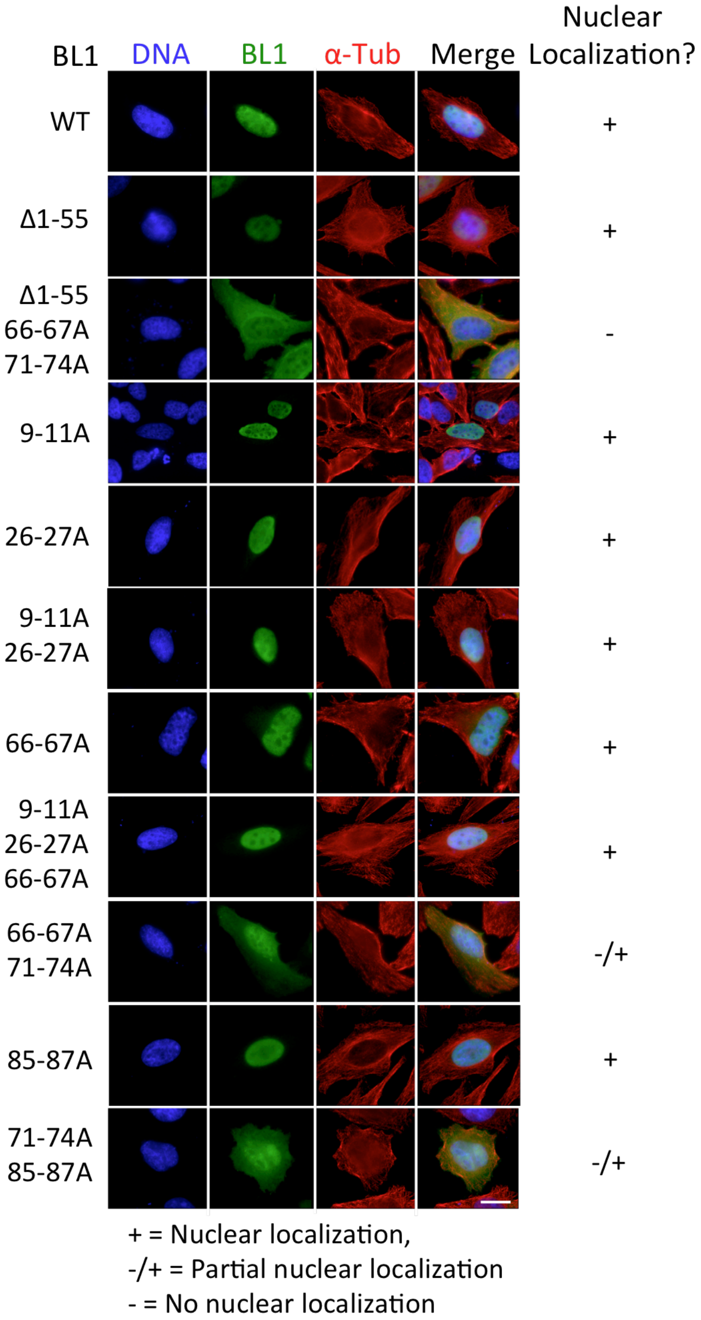

Supplemental Fig. S4. **KATNBL1 localization to the nucleus during interphase requires an N-terminal nuclear localization sequence.** Immunofluorescence microscopy of cells transfected with wildtype (WT) GFP-KATNBL1, GFP-KATNBL1 NLS1 and NLS2 mutants, and GFP-KATNBL1 truncation mutants as indicated for 24 hours. Cells were fixed with paraformaldehyde and stained with Hoechst 33342 to detect the DNA, anti-α-tubulin antibodies to detect microtubules, and anti-GFP antibodies to detect GFP-KATNBL1 localization. + indicates nuclear localization, +/- indicates partial nuclear localization and – indicates no nuclear localization. Bar= 5μm. See Fig. 5 for the localization of additional mutants. For a list of primers used for KATNBL1 mutagenesis see supplemental Table S3.

Supplemental Tables

Supplemental Table S1. **Summary of the Katanin interactome (Katan-ome).** Summary of LAP-KATNA1, KATNAL1, KATNAL2, KATNB1 and KATNBL1 interacting proteins identified by biochemical tandem affinity purifications and mass spectrometry analyses from mitotic cells. List includes protein name, UniProt accession number, number of peptides identified, the percent protein coverage and a description of each of the identified proteins.

Supplemental Table S2. **Spectra of one peptide IDs in the Katan-ome.** Spectra of one peptide IDs in the LAP-KATNA1, KATNAL1, KATNAL2, KATNB1 and KATNBL1 mass spectrometry analyses from mitotic cells.

Supplemental Table S3. **List of primers used to mutate KATNBL1.** List of primers used to generate KATNBL1 nuclear localization sequence mutants and truncation mutants.
