# Supplementary material for: Proteomic Analysis of the Mammalian Katanin Family of Microtubule-severing Enzymes Defines Katanin p80 subunit B-like 1 (KATNBL1) as a Regulator of Mammalian Katanin Microtubule-severing
Source: Mol Cell Proteomics. 2016 Feb 29;15(5):1658–69. doi: 10.1074/mcp.M115.056465 (PMC4858946; doi:10.1074/mcp.M115.056465)
Supplement: Supplemental Data [file 10.1074_M115.056465_mcp.M115.056465-4.docx]

| **Construct** | **Seq Name** | **Annealing Sequence** | **Seq 5’ to 3’** |
| --- | --- | --- | --- |
| KATNBL1 WT | KATNB2.For | ATGGCATCAGAAACCCACAATGTT | GGGGACAAGTTTGTACAAAAAAGCAGGCTTCGAAGGAGATAGAACCATGGCATCAGAAACCCACAATGTT |
|  | KATNB2.RevS | ATGTAACTGTAATAAATAA | GGGGACCACTTTGTACAAGAAAGCTGGGTCCTAATGTAACTGTAATAAATAA |
| KATNBL1 Δ1-55 | KATNB2_Δ1-55.For | GGACAAACTGTGAAAAGCCC | GGGGACAAGTTTGTACAAAAAAGCAGGCTTCATGGGGGGACAAACTGTGAAAAGCCC |
|  | KATNB2.RevS | ATGTAACTGTAATAAATAA | GGGGACCACTTTGTACAAGAAAGCTGGGTCCTAATGTAACTGTAATAAATAA |
| KATNBL1 1-55 | KATNB2.For | ATGGCATCAGAAACCCACAATGTT | GGGGACAAGTTTGTACAAAAAAGCAGGCTTCGAAGGAGATAGAACCATGGCATCAGAAACCCACAATGTT |
|  | KATNB2_1-55.RevS | CAA CTG TTC TAT TTA TGT AAG CAG CC | GGGGACCACTTTGTACAAGAAAGCTGGGTGTCA CAA CTG TTC TAT TTA TGT AAG CAG CC |
| KATNBL1 1-69 | KATNB2.For | Primer box - B3 |  |
|  | KATNB2_1-69.RevS | GAT CAC TTT ACG AAG TTT ATC TGG GC | GGGGACCACTTTGTACAAGAAAGCTGGGTG TCA GAT CAC TTT ACG AAG TTT ATC TGG GC |
| KATNBL1 1-98 | KATNB2.For | Primer box - B3 |  |
|  | KATNB2_1-98.RevS | TGC CAT GTC ACA GCC C | GGGGACCACTTTGTACAAGAAAGCTGGGTG TCA TGC CAT GTC ACA GCC C |
| KATNBL1 K9A/K10A/R11A  Step 1 | B2_K9A | ATGGCATCAGAAACCCACAATGTTGCAAAACGGAACTTTTGTAATAAGATTGAGG | |
|  | B2_K9A-r | CCTCAATCTTATTACAAAAGTTCCGTTTTGCAACATTGTGGGTTTCTGATGCCAT | |
| KATNBL1 K9A/K10A/R11A  Step 2 | B2_K9A/K10A/R11A | AATGAAATGATCCTCAATCTTATTACAAAAGTTCGCTGCTGCAACATTGTGGGTTTCTGATGCCAT | |
|  | B2_K9A/K10A/R11A-r | ATGGCATCAGAAACCCACAATGTTGCAGCAGCGAACTTTTGTAATAAGATTGAGGATCATTTCATT | |
| KATNBL1 R26A/K27A | B2_R26AK27A.For | CTT CAT GTT CTT ATT AGT GAA ATT AGA GAT CTT TGC TGC AGG AAG ATC AAT GAA ATG ATC CTC AAT CTT ATT A | |
|  | B2_R26AK27A.Rev | TAA TAA GAT TGA GGA TCA TTT CAT TGA TCT TCC TGC AGC AAA GAT CTC TAA TTT CAC TAA TAA GAA CAT GAA G | |
| KATNBL1 R66A/K67A | B2_R66A/K67A | GAACTTTCTTTCTGCGATAGATCACTGCAGCAAGTTTATCTGGGCTTTTCACAGTTTGTC | |
|  | B2_R66A/K67A-r | GACAAACTGTGAAAAGCCCAGATAAACTTGCTGCAGTGATCTATCGCAGAAAGAAAGTTC | |
| KATNBL1 R71A/R72A/K73A/K74A  Step 1 | B2_R71A/R72A | GGAAAGGGATGATGAACTTTCTTTGCGGCATAGATCACTTTACGAAGTTTATCTGG | |
|  | B2_R71A/R72A-r | CCAGATAAACTTCGTAAAGTGATCTATGCCGCAAAGAAAGTTCATCATCCCTTTCC | |
| KATNBL1 R71A/R72A/K73A/K74A  Step 2 | B2_R7172A/K7374A | CTGTAACAAGGATTTGGAAAGGGATGATGAACTGCCGCTGCGGCATAGATCACTTTACGAAGTTTATCTGGGCTTTTCACAGT | |
|  | B2_R7172A/K7374A-r | ACTGTGAAAAGCCCAGATAAACTTCGTAAAGTGATCTATGCCGCAGCGGCAGTTCATCATCCCTTTCCAAATCCTTGTTACAG | |
| KATNBL1  R85A/K86A/K87A  Step 1 | B2R85AK86Astep1.For | CCC CAC TTC CAG GGG ACT GTT TTG CTG CGT AAC AAG GAT TTG GAA AGG GAT | |
|  | B2R85AK86Astep1.Rev | ATC CCT TTC CAA ATC CTT GTT ACG CAG CAA AAC AGT CCC CTG GAA GTG GGG | |
| KATNBL1  R85A/K86A/K87A  Step 2 | B2R85AK86Astep2.For | CCC CAC TTC CAG GGG ACT GTG CTG CTG CGT AAC AAG GAT TTG GAA AGG GAT | |
|  | B2R85AK86Astep2.Rev | ATC CCT TTC CAA ATC CTT GTT ACG CAG CAG CAC AGT CCC CTG GAA GTG GGG | |

Table S3
